# A right to remoteness? A missing bridge and articulations of indigeneity along an East Siberian railroad

**DOI:** 10.1111/1469-8676.12648

**Published:** 2019-06-06

**Authors:** Peter Schweitzer, Olga Povoroznyuk

**Affiliations:** ^1^ Department of Social and Cultural Anthropology University of Vienna Universitaetsstrasse 7 Vienna Austria

**Keywords:** remoteness, indigeneity, culture, bridge, Siberia, éloignement/isolement, indigénéité, culture, pont, Sibérie

## Abstract

The Soviet Union and its successor states have been avid supporters of a modernisation paradigm aimed at ‘overcoming remoteness’ and ‘bringing civilisation’ to the periphery and its ‘backward’ indigenous people. The Baikal–Amur Mainline (BAM) railroad, built as a much‐hyped prestige project of late socialism, is a good example of that. The BAM has affected indigenous communities and reconfigured the geographic and social space of East Siberia. Our case study, an Evenki village located fairly close to the BAM, is (in)famous today for its supposed refusal to get connected via a bridge to the nearby railroad town. Some actors portray this disconnection as a sign of backwardness, while others celebrate it as the main reason for native language retention and cultural preservation. Focusing on discourses linking the notions of remoteness and cultural revitalisation, the article argues for conceptualising the story of the missing bridge not as the result of political resistance but rather as an articulation of indigeneity, which foregrounds cultural rights over more contentious political claims. Thus, the article explores constellations of remoteness and indigeneity, posing the question whether there might be a moral right to remoteness to be claimed by those who view spatial distance as a potential resource.

## Un droit à l’isolement ? Un pont manquant et des articulations d’indigénéité le long d’un chemin de fer en Sibérie orientale

L’Union soviétique et ses États successeurs ont été de fervents partisans d’un paradigme de modernisation visant à « surmonter l’éloignement » et à « amener la civilisation » à la périphérie et à son peuple indigène « arriéré ». Le chemin de fer Magistrale Baïkal–Amour (la BAM), construit en tant que projet prestigieux très médiatisé du socialisme tardif, en est un bon exemple. La BAM a impacté les communautés autochtones et reconfiguré l’espace géographique et social de la Sibérie orientale. Notre étude de cas se focalise sur un village d’Evenki situé assez près de la BAM, célèbre aujourd’hui pour son refus supposé de se connecter par un pont à la ville ferroviaire à proximité. Certains acteurs considèrent cette déconnexion comme un signe de retard, tandis que d’autres la célèbrent comme la raison principale de la préservation de la langue maternelle et de la culture. Se focalisant sur des discours reliant les notions d’éloignement/isolement et de revitalisation culturelle, l’article plaide en faveur d’une conceptualisation du récit du pont manquant, non comme le résultat de résistance politique, mais plutôt comme une articulation de l’indigénéité mettant en avant des droits culturels plutôt que des revendications politiques plus controversées. L’article examine ainsi des constellations d’éloignement et d’indigénéité soulevant la question d’un droit moral à l’isolement qui serait revendiqué par ceux qui conçoivent la distance spatiale comme ressource potentielle.

## Introduction

Alongside the BAM,^1^ a relatively new railroad traversing eastern Siberia north of Lake Baikal, the small and predominantly indigenous community of Ust’‐Nyukzha has gained notoriety for supposedly rejecting – back in the 1980s – the construction of a river bridge that would have provided a year‐round connection to the railway line and beyond. While ‘what actually happened’ more than 30 years ago remains unclear from the oral history and written records, it is the prominence of the ‘missing bridge’ in local and regional discourses of today that has prompted this article.

The history of infrastructure and modernisation projects is punctuated by protests against them (see, e.g. Chu 2014; Schüler 2017; von Schnitzler 2016). There are, however, no documented traces of protest or ‘resistance’ (see, e.g. Gellner 2007) against the railroad in question. While this can be understood as a result of the authoritarian and non‐transparent political culture of the Soviet Union at the time, reports about environmental protests against the degradation of nearby Lake Baikal (see, e.g. Rainey 1991; Zaharchenko 1990; Ziegler 1987) confirm that any kind of ‘movement’ would certainly have reached the attention of western scholars. The notion of ‘refusal’, on the other hand, recently propagated by Audra Simpson (2014, 2017) in the context of North American (and Australian) indigenous studies, cannot be as easily dismissed by the absence of ‘protest’ as defined by western expectations. At the same time, the political subjectivities of indigenous individuals and communities in Soviet and post‐Soviet contexts seem to be quite different from the Mohawk and others written about by Simpson.

As mentioned above, what happened in the 1980s is not really of central relevance here. Instead, we are interested in the social contexts in which the missing bridge is used today. Our title question obviously alludes to Henri Lefebvre's famous phrase of the ‘right to the city’ (Lefebvre 1996 [1968]). While his text, originally published in the 1960s, was a battle cry against the deteriorating cities of his day, subsequent users of the phrase rediscovered its applicability under conditions of neoliberalism and increased privatisation of urban spaces (see, e.g. Harvey 2008). As Attoh (2011) has pointed out, however, it remains unclear in many of these usages what kind of right ‘the right to the city’ is. In the context of the Russian Federation (or anywhere else, to our knowledge), there is certainly no legal right to remoteness, as such.

Thus, our article explores constellations of remoteness and indigeneity, posing the provocative question whether there might be a moral right to remoteness. This entails a view of remoteness as a potential resource to some and as an obstacle to others. At the same time, we acknowledge multiple meanings (spatial, cultural, etc.) of remoteness. Thus, our investigation will follow how different notions of remoteness are being co‐constructed in the story of the missing bridge. This leads to questions such as how these notions of remoteness (and connectivity) are being imagined and experienced by different groups in the region, what outsider and insider perspectives on the missing bridge are, and how notions of otherness are being merged with spatial concepts of distance and disconnection.

## Remoteness, modernisation and articulations of indigeneity

In concert with the editors of this special issue, we see ‘remoteness’ not as a primordial condition but as a socio‐spatial concept (Hussain 2015) that is relational and relativising and thereby constantly open to reconfigurations. As Martin Saxer points out, remoteness should also be seen in conjunction with connectivity, and ‘remoteness is not only a relational condition, but in many places also a relatively recent one’ (2016: 110).

At the same time, ‘remoteness’ has received too little theoretical or conceptual attention within anthropology. While some notions of spatial distance and ‘out‐of‐the‐way‐ness’ dominated – consciously or unconsciously – the selection of anthropological field sites in early years of the discipline, neither the ontological nor the epistemological qualities of ‘being remote’ have typically made it to the level of research questions. On the contrary, as the examples of the emergence of urban, global, transnational and other kinds of anthropologies show, ‘remote’ served as the supposedly natural starting point of anthropologists’ choices of research areas. While there have been manifestos for urban anthropology, or global or transnational connections, seemingly nobody felt the need to produce them for ‘remote anthropology’ or an ‘anthropology of remoteness’.^2^

Since the re‐publication of Edwin Ardener's seminal article on ‘remote areas’ in 2012, an increase in theoretical interest in ‘remoteness’ within anthropology has become noticeable. After all, when Ardener's article was first published in 1987, it was in an edited volume entitled *Anthropology at home* – thus, one might conclude that the anthropological conceptual work on ‘remoteness’ started – and was quickly abandoned again – in reaction to anthropologists practising other forms of anthropology than what had been the unquestioned – and largely unspoken – rule of where the discipline should be practised. Ardener's article – and even more so the collection of articles entitled ‘Remote and edgy’ and published in 2014 (Harms *et al*. 2014) – made it clear that an anthropological engagement with the notion of ‘remoteness’ cannot be limited to spatial dimensions, though of course spatial elements do matter: spatial distance (from political and administrative centres) and difficult access have been important to individuals and groups throughout history, either to protect their ‘unlicensed’ faith, hide their activities not approved by the centre or avoid higher property taxes, to name just a few possible reasons. James Scott's insistence that some marginal groups have chosen their (remote) location to maintain their autonomy (Scott 2009) fits well with our understanding that remoteness can be a resource and carry positive value to some groups at certain points in time.

As our regional focus is defined by a railroad – that is, by a form of transportation infrastructure – it might be reasonable to assume that the ultimate goal of the (state‐financed and directed) endeavour was to ‘overcome remoteness’ (for raw materials, goods and people). While this is a classical goal of development ideologies (Arce and Long 2000; Li 2007), it is also a way of depoliticising decisions of social and spatial significance (Ferguson 1994). Railroad construction in general can be seen as a prototypical modernisation project involving a number of linked ideological, infrastructural, political, socio‐economic and cultural processes (Kaschuba 2004; Schivelbusch 2000). Unlike European projects belonging mostly to the 19th century, Russian/Soviet railroad projects have been primarily a feature of the 20th century, implementing ideologies and policies of ‘high modernism’ (Scott 1998).

The Soviet industrialisation programme of ‘mastering the North’, with its underlying modernist idea of human dominance over nature, constructed the northern frontier territories as hostile and their local population as uncivilised and backward. In this context, the definition of remote carried mostly negative connotations and was interpreted as being synonymous with ‘uncivilised’, ‘backward’ or ‘Other’. Thus, modernity and remoteness were officially and discursively constructed as two poles of the modernisation paradigm (cf. Hussain 2015).

Discussions about the missing bridge are primarily situated within the discursive space of indigeneity, by indigenous and non‐indigenous speakers alike. Without entering the seemingly endless conversation on ‘what is indigeneity’, we follow Tania Murray Li's (2000) usage of ‘positioning’ and ‘articulation’ in the context of indigenous identities. The term ‘articulation’, which has a long Marxist genealogy reaching from Gramsci to Althusser, is adopted by Li from the writings of Stuart Hall. Hall's notion of articulation acknowledges that distinct elements can be combined, need not be reduced to an ultimate cause and are relations of ‘no necessary correspondence’ (Hall 1996: 14). This enables Li – and us – to speak of indigenous identities as ‘positionings’ and not as primordial essences. Thus, our case of the never‐built bridge and the different usages of remoteness it has triggered are seen as articulations of indigeneity, and as positionings in relation to these articulations.

## Regional preliminaries

### The history of the Baikal–Amur Mainline (BAM) railroad

‘The BAM construction breathed new life into this taiga region’ were the dramatic words used in a report by the district administration in Tynda (Pasport 1990). The region suddenly found itself at the epicentre of a gigantic construction process. The major route of the BAM intersects with the Amur–Yakutsk Mainline (AYaM), which provides a connection to the Trans‐Siberian railroad, in the district centre of Tynda. The latter turned not only into a transportation hub but also the ‘capital city’ of the region hosting the administration of the BAM. The impacts of the BAM on local mobility patterns, traditional industries, indigenous culture and language were comparable to, if not exceeding, the magnitude of the collectivisation reforms that swamped the region and its people in the early Soviet period.

The BAM is among the longest and northernmost railroads in the world and the largest industrial and modernisation projects of the late socialist period of the Soviet Union. It traverses the Northern districts of five regions in Eastern Siberia and the Russian Far East, with its longest side branch, the AYaM, stretching to the southern part of the Republic of Sakha (Yakutiya).^3^ According to the 2010 All‐Russian census, the largest cities along the BAM include Komsomol'sk−na‐Amure (263,906 people), Neryungri (61,747 people), Ust’‐Kut (45,375 people), Tynda (36,275 people), Tayshet (35,485 people) and Severobaykal'sk (24,929 people), while more typical settlements have a population between 300 and 4,000 residents. Most of the indigenous Evenki people live in so‐called ‘national villages’ (*natsional'nye poselki*),^4^ located off the railroad and sometimes off roads.

While its history starts with early construction projects dating back to the 19th century, the majority of the railroad was built between 1974 and 1984. The BAM as a continuation of the Soviet modernisation project had a mission of ‘bringing civilisation to remote corners of the country’. In this modernisation paradigm, local, primarily indigenous, people were imagined just as being remote – other, backward and underdeveloped.^5^ Under the conditions of late Soviet socialism and its planned economy, mass labour mobilisation stimulated by ideological propaganda and material benefits attracted migrants to the BAM construction. The local population was retained in the kolkhoz and public sectors, while the construction works were to be realised by a labour force recruited from across the Soviet Union.

The railroad affected indigenous Evenki and other Tungusic‐speaking peoples living in the region in various ways. While nomadic reindeer herders and hunters suffered degradation or a loss of traditional lands and domestic reindeer and game, the village population was exposed to intense cultural contact and uneven exchange with the inflowing migrants. Evenki involvement in the railroad construction was limited to unskilled work (porters, stone dressers, wood cutters) at the initial stages of the project and trade (supply of traditional produce) between local kolkhozes and construction companies. Higher salaries, a number of social benefits and the labour prestige of the BAM builders created a social gap between the migrants recruited for the railway construction from across the Soviet Union (Ward 2009) and the local population consisting primarily of indigenous Evenki people. This social and cultural boundary between indigenous people (*aborigeny*) and the BAM builders (*bamovtsy*) was also reproduced through a settlement pattern where indigenous villages were spatially separated from the settler towns emerging along the railroad. An image of Evenki communities as remote and marginal, both in spatial and social terms, quickly spread among the *bamovtsy*.

### Post‐socialist reconfigurations of space

*Perestroika* and the subsequent dissolution of the Soviet Union challenged the modernist paradigm in general and the BAM myth in particular. Political changes and the withdrawal of state subsidies in the 1990s reconfigured geographical and social spaces through an infrastructural collapse and a ‘return of remoteness’. The degradation and ruination of roads and railroad tracks, as well as the discontinuation of regular air connections became widespread phenomena across the sparsely populated areas of the North (Campbell 2003). For example, after the cancellation of air flights to the Evenki settlement of Sredniy Kalar, its residents found themselves in forced isolation (Povoroznyuk 2011). The parallel curtailing of support for the social infrastructure of the settlements on and off the BAM – which had been provided by the Soviet state and was taken for granted (Humphrey 2003) – deprived local residents of vitally important medical, banking and other services. Currently, one has to travel the distance of over 300 km from Yuktali to the district centre of Tynda in order to see a doctor. This kind of remoteness seems to be growing, especially among the ‘surplus’ population of BAM builders left after development (cf. Li 2017).

The socio‐economic crisis of the 1990s provoked public criticism of the BAM project due to its high construction and maintenance costs and environmental impacts and raised the issue of assimilation of indigenous people. The environmental and indigenous rights movements grew in Russia from the mid‐1980s to the early 2000s inspired by similar international movements. While some of these globally operating organisations at least partially relied on essentialist concepts and constructed a particular type of indigeneity (Ghosh 2006), they opened a public space for alternative discourses and perceptions of remoteness. For the first time, indigenous NGOs, cultural leaders and their supporters spoke about remoteness in the context of tradition and modernity (Pika 1999), territorial rights (Fondahl *et al*. 2001) and sustainable development.

While no uniform counter‐discourse to modernising concepts was formed during that short time period, the notion of remoteness acquired more positive connotations: from being out of the way and/or abandoned to a symbolic resource helping to protect traditional lands and indigenous culture. In the 1990s, indigenous activists strategically used the concept to claim their land rights and to be physically remote from the industrial infrastructures of the BAM and resource extraction sites.

Currently, the Russian state tends to re‐appropriate indigenous lands and recall their special territorial rights altogether. In this situation, cultural rights often remain the single vestiges of indigeneity and appear as compensation for political marginalisation. While contacts with most foreign indigenous organisations have become more difficult, cultural exchanges with Evenki communities in China are on the rise. A recently launched state railroad modernisation programme, BAM‐2, is, in essence, a continuation of Soviet construction and development plans, both on ideological and material levels (Ssorin‐Chaikov 2016). Although carried out in a different socio‐economic and geopolitical context, it re‐enchants the local communities with promises of modernity, connectivity, speed and socio‐economic development (cf. Harvey and Knox 2012). These new hopes, however, clash with physical distance and economic austerity. While some, mostly young, Evenki work ‘on the rails’, others are concerned about potentially adverse effects of these new state modernisation efforts and the increased connectivity that might result from them.

## The case of Ust’‐Nyukzha^6^

### Historical background

We have a village that is accessible exclusively by train. Last spring, they wanted to cancel the train Komsomol'sk‐na‐Amure–Tynda, but people protested because it is impossible to access the village at any time, but in winter. Ust’‐Nyukzha is also currently immobile: the freeze‐up starts and they cannot cross the river. But they don't want to build a bridge, because the bridge brings cars, Russian hunters and a supply of vodka and other bad products. When the issue of the bridge was discussed and the money for its construction was allocated, they [the local residents] decided not to build it. … good for them! (Interview: NFK, Tynda, 2013)

We first heard about this widely circulating story from a specialist on indigenous affairs in Tynda, the administrative centre of Tyndinskiy District. The story relates that one day during the peak of the BAM construction in the 1980s at a meeting of residents of Ust’‐Nyukzha village, a wide‐reaching decision was made – not to build a bridge that would have connected the village with its twin settlement Yuktali. Furthermore, albeit undocumented, the story of the missing bridge seems to interrelate spatial disconnection and cultural otherness of the village: the missing bridge seems to make Ust’‐Nyukzha culturally central for Evenki people, but remote for the rest of the population.

The ‘national village’ of Ust’‐Nyukzha was founded as a trading post (*faktoriya*) in 1923. It is home to a majority population of Evenki, some Sakha families, descendants from Russian Old Settlers, as well as Soviet migrants of diverse (primarily Slavic) backgrounds, who came with the construction of the BAM. The Nyukzha River (a tributary of the Olekma) separates the village from the nearby railroad town of Yuktali that lies only 7 km away. Yuktali was founded by BAM builders in 1976 and was first called Ust‐Nyukzha‐2. ‘Old’ (Ust’)‐Nyukzha, as it is still called by locals on both sides of the river, stands out among other national villages for its ‘traditional’ nomadic lifestyles, Evenki cultural festivals, high native language retention rates and a nomadic school, one of a few of its kind in the Russian North.

The local Evenki belong to a culturally distinct territorial group of this widely dispersed ethnic group, whose nomadic ancestors hunted and herded their reindeer in the valleys of Vitim and Olekma rivers before collectivisation. Large herds of 400–500 domestic animals have been grazing on the pastures that are presently part of the Republic of Sakha (Yakutiya) and northern Zabaykal'skiy Region. Collectivisation boosted the sedentarisation of nomads and the growth of the village. In the early 1930s, the first school was built, the local administration was formed and soon thereafter the collective farm *Lenin Okton* (meaning ‘Lenin's Way’ in Evenki) was established (Pasport 2012: 1–2).

Throughout the Soviet period, hunting remained an important indigenous subsistence activity, despite the fact that state development plans focused on reindeer herding. In addition to the so‐called ‘traditional industries’, new agricultural branches were introduced. Fur farming, cattle breeding and horticulture became part of collective farming in Ust’‐Nyukzha, like elsewhere in the Soviet North (Grant 1995; Humphrey 1983). By the 1970s, Ust’‐Nyukzha had its own hospital, a school and a boarding school, a kindergarten, a shop and a post office. Yet, it was almost exclusively the seasonal ice‐road Dhzeltulak–Ust’‐Urkima–Ust’‐Nyukzha that made the connection with the district centre in Tyndinskiy possible. While communication and the supply of goods by trucks was possible in winter, no regular connection with the village was available during other seasons, except for occasional helicopters and airplanes sent to this ‘deep’ taiga area by geological expeditions (Tugolukov 2005: 229).

The BAM construction has led to drastic demographic, socio‐economic and cultural changes. The laying of tracks, the construction of haul roads and the logging practised by Korean companies affected pastures and hunting grounds and changed the seasonal sequence of animal migrations. Evenki residents in Ust’‐Nyukzha still recall the following:

Elders were first naturally against the BAM – they were afraid that newcomers would hunt the animals to extinction … but then had to accept it. (Interview: GAA, 2017)

The local collective farm in Ust’‐Nyukzha got involved in the supply of so‐called ‘traditional products’ (mostly reindeer meat) to the BAM builders. The construction companies opened a ‘BAM shop’ in the village: there the local residents could acquire – through purchase or through the exchange of their products – food items and other goods the BAM builders had received from Moscow and from socialist countries (such as Bulgaria, East Germany, Hungary, etc.). Following the official end of the BAM construction period and the beginning of the post‐Soviet socio‐economic crisis, collective herding declined and, in 2001, the farms were reorganised into *obschchiny –* indigenous non‐commercial land use enterprises.

### Living without the bridge today^7^

Currently, Ust’‐Nyukzha is a ‘national village’ with a population of 649 residents, 414 of whom are Evenkis. Yuktali, on the opposite side of the river, is a typical medium‐size town along the BAM with a predominant *bamovtsy* population (1,615 in 2016), urban housing and infrastructure (Pasport 2017b). In contrast to mass population outflow from Yuktali,^8^ the local population in Ust’‐Nyukzha has been growing due to a high birth‐rate and a low level of outmigration (Pasport 2017a). The main spheres of employment of village residents include transportation (most importantly, the railroad company servicing the BAM), communal services, education and trade (in 2017, the community had eight small shops). Many villagers keep cattle, horses, pigs and poultry at their individual plots for subsistence. Ust’‐Nyukzha is home to a few nomadic Evenki enterprises. Eight reindeer‐herding enterprises with a total reindeer head count of 1,930 have been registered within the municipality's boundaries and more indigenous families are informally involved in herding. Hunting (especially for fur) is widely practised by both indigenous and non‐indigenous residents.

Despite the fact that Ust’‐Nyukzha has been affected by the BAM, research conducted by Soviet and, later, Russian anthropologists tends to emphasise the cultural and linguistic distinctiveness of the community *vis‐à‐vis* other indigenous villages in the region. For example, Ust’‐Nyukzha has the highest rates of indigenous language identification and retention in the Tyndinskiy District. According to one survey conducted in 1989, 81.7% of the Evenkis of Ust’‐Nyukzha considered the Evenki language to be their native language (Turaev 2004). Another feature that distinguishes the village in the field of indigenous education and language retention is a secondary school with a strong emphasis on teaching the Evenki language and traditional industries. The innovative project of a nomadic elementary school for reindeer herders’ children was recently successfully introduced by a French anthropologist doing long‐term research and living in the community (Lavrillier 2013). The nomadic school for indigenous children (one among very few similar initiatives in Russia), combined with the fact that in 2012 the village hosted an Evenki festival ‘*Bakaldyn*’, has added to the popularity of Ust’‐Nyukzha as a ‘hub’ of indigenous culture.

While there is no visible difference in lifestyles and occupations between indigenous and non‐indigenous residents of the village, Evenki identities become articulated in discourses about indigenous rights and, more recently, about cultural revitalisation. Cultural events (such as the Evenki festival mentioned above), as well as negotiations over native language and education initiatives and ethno‐tourism investment programmes, provide the public space for indigenous leaders to voice their concerns and claim their rights.

Their consciousness has been re‐awakened: they will not change their ethnic identity as they would have done earlier when they were not using or even willing to use Evenki language. When the politics of the cultural revitalisation of Evenki people, Evenki rites and similar started … they got more interested in their own life, in the life of their people and in their language. (Interview: KSA, Tynda, 2016)

The missing bridge is one of the factors that allows Ust’‐Nyukzha to be officially categorised as a ‘remote’ and ‘hard‐to‐access region of North’ (*otdalennyy*,* trudnodostupnyy rayon Severa*) (cf. Kuklina and Holland 2018). Yet, the missing bridge symbolises not only physical disconnection and separation but also the border between indigenous and non‐indigenous communities representing two different cultural worlds on opposite sides of the river. Thus, it brings together different meanings and constellations of remoteness, otherness and indigeneity.

Most local residents understand remoteness in spatial (*otdalennost’*), geo‐political (being far away from administrative centres) and socio‐economic (being unprofitable, needing subsidies) terms. The similar words ‘disconnected’ or ‘detached’ (*otorvannye*) were also used by our interlocutors to broadly refer to communication gaps (lack of roads or mobile phone and Internet connection). Remoteness as a socio‐cultural category opens a much wider space for interpretation, ranging from ‘isolation from civilisation’ (*‘otorvannost’ ot tsivilisatsii’*) to ‘wilderness’ (*dikost’, dikaya priroda*), to, finally, ‘cultural distinctiveness’ (*obosoblennost’*). Recently, there has been a shift from negative to positive interpretations of remoteness. In the context of cultural revitalisation, this shift is an expression of the changing positionality of indigenous people and articulations of indigeneity (Li 2000) in post‐Soviet Russia.

### Remoteness: a gaze from the outside

Our field observations and interviews speak to the relational and contextual qualities of the notion of remoteness. When analysing the concept of remoteness, one should take into account its multiple meanings and uses informed by the interplay between experiential and discursive, physical and symbolic domains and factors such as place of residence, ethnicity and occupation.

The range of attitudes towards remoteness among our interviewees allows us to make a distinction between outside and inside perspectives on the missing bridge. While the Ust’‐Nyukzha case is widely discussed on a regional scale, including the district centre of Tynda, the most critical opinions are voiced in Yuktali on the opposite side of the river. Yuktali plays here the role of an urbanised centre in relation to its remote ‘periphery’. The modern infrastructures and amenities of Yuktali are often juxtaposed with rural life and the lack of comfort in the nearby rural settlement. The mere existence of Ust’‐Nyukzha is seen as depending on the BAM and supplies coming from Yuktali:

Ust’‐Nyukzha would have died long ago in the 1990s. Earlier we had a cooperative consumer association here. They supplied food from Tynda on trucks by an ice road … And who would have made an ice road for them; who would have supplied them (Ust’‐Nyukzha residents) now? (Interview: NVM, Yuktali, 2017)

The post‐Soviet decline of communal infrastructure and social services currently experienced in Yuktali, as well as the missing bridge to Ust’‐Nyukzha, are seen as challenging. Many residents in Yuktali, which still consists primarily of *bamovtsy*, express bewilderment toward Ust’‐Nyukzha's refusal of the bridge:

I am not sure if they [the residents of Ust’‐Nyukzha] admitted their mistake. Bamovtsy wanted to build a bridge there, but they refused and said: ‘No, [unwanted, non‐local] people will be coming here.’ I am not even sure what kind of repercussions they might have to face now. (Interview: NVM, Yuktali, 2017)

Other residents of Yuktali, who came to work for the BAM as doctors, teachers and kindergarten nurses, share a similar modernisation perspective regarding the missing bridge. When talking about the remoteness of Ust’‐Nyukzha, the case of the bridge pops up as an example of an unreasonable, emotion‐driven decision against the comforts of ‘civilisation’. Even if the person whose words are cited below is a descendant of a mixed Russian–Evenki family and the wife of a non‐Russian BAM builder, she clearly blames the ‘uncivilised’ and ‘uncultured’ behaviour of the Evenkis for their decision:

When Evenkis were allocated money, they said: ‘Why do we need a bridge? Bamovtsy would come and harvest our berries ….’ Now, they should have been happy to have one: the hospital is here and they have to come by boats. I feel sincerely sorry for them. It might be OK for an adult, but what if you would need to come with a baby? What if there is floating ice? (Interview: IKK, Yuktali, 2017)

### Remoteness from within

While the polyphony of voices and deliberations among local residents makes an insider/outsider dichotomy challenging, spatial and symbolic remoteness is experienced and perceived differently in Ust’‐Nyukzha than in Yuktali. Which value an individual assigns to remoteness depends a lot on the social characteristics (such as age, occupation, ethnicity) of that person. *Bamovtsy* or those education and healthcare professionals who came to the village with the BAM construction have typically a more negative view of remoteness. Indigenous and pre‐BAM settlers, on the other hand, see remoteness rather as a resource than a negative quality. While the majority of villagers do not feel remote or uncomfortable without a road bridge, they are not totally opposed to the BAM, development or ‘modern’ life with its promises, including certain forms of connectivity and mobility.

The missing bridge is the most tangible symbol of remoteness and stands for disconnection from ‘the Big Land’.^9^ The absence of a permanent road connection makes Ust’‐Nyukzha fully accessible for ground transportation and large‐scale supply only during the winter season. It is only the publicly‐run boat that keeps the village connected to neighbouring Yuktali and the BAM from mid‐May through September. A recent attempt to introduce a hovercraft did not pay off due to high maintenance costs, according to the local administration. The high costs of fuel and technical service required by the vehicle could not be covered by local users without state subsidies. Therefore, the administration rents it out to private entrepreneurs and calls it back to public community service only in emergency situations. In cases of fire, health emergencies, deaths and births, helicopters can also be called from Chul'man, the nearest airport in the southern part of the Republic of Sakha (Yakutiya).

The local administration of the village has a clear pro‐development vision. The head of the administration, a former *bamovets* who settled in the village, sees the absence of a bridge and the disconnection from the BAM as a major problem and as a sign of socio‐economic decline and abandonment. His nostalgic reference to the golden age of the BAM construction is, therefore, not surprising:

Of course, the BAM boosted the economic development of the district, first of all, in terms of the transportation infrastructure. It gave an opportunity to supply products and materials at any time, not only by the winter road. Yet, we still remain a village with a limited supply period. As you see, we live on an island … No one needs us – the railroad doesn't need us because we are lying out of its way. We used to have a collective farm and supply meat on an industrial scale; we had pig and fur farms, they were interested in us. Now we don't produce or supply anything except for the workforce – young people who work at the railroad. This is the main reason of our impoverished existence … (Interview: AVM, Ust’‐Nyukzha, 2017)

The spatial remoteness of Ust’‐Nyukzha may indeed present challenges for local residents. The periods of freeze‐up and floating ice make the use of public and private boats a highly risky endeavour. Yet, some residents have to resort to it, especially in cases when they need to get to their work at the railroad headquarters or urgently see a doctor in Yuktali or Tynda. A few local residents currently work ‘at the rails’ or at the new construction project launched within the ‘BAM‐2’ programme. They have to regularly commute between the two settlements and risk their lives crossing the river during freeze‐up and spring melt, something they consider routine business. Likewise, pregnant women need to get across the river in any season to deliver their children in the hospital, which has recently moved from Yuktali to Tynda. For these pragmatic reasons, a public boat service and a pedestrian bridge appear to be reasonable solutions to many, while a road bridge is still mostly unwanted.

Evenki reindeer herders and hunters have a more or less straightforward interpretation of remoteness as a positive and desirable condition. Their concept of space is based on differentiation between the ‘peace’ of the taiga, the ‘headache’ of the village, and the loud and aggressive interventions from the ‘outside’. Their wish to be physically remote from the BAM is rooted in negative experiences of the impacts of the railroad. Reindeer herders whose pastures are traversed by the railroad tracks continue to suffer losses. Their domestic animals often get scared or injured when crossing the rails or fall prey to wolves and other predators attracted by waste food thrown out of passenger trains [Interviews: SAK, OKA, Ust’‐Nyukzha, 2017].

Thus, the lifeworlds of Ust’‐Nyukzha residents demonstrate that connectivity and remoteness co‐constitute each other. Local residents do interact with the ‘outside’ world, an observation already made by Ardener in other contexts (‘remote areas are in constant contact with the world’; Ardener 2012: 528–9). They see the advantages of connectivity (e.g. in terms of their own mobility), but feel vulnerable to certain forms of it (e.g. ‘invasion’ from outside and negative impacts of the railroad). The following interview with an Evenki leader and NGO activist talking about the importance of the railroad illustrates that spatial remoteness and disconnection of Ust’‐Nyukzha are relative and relational:

It's good that they opened a railroad for us [in the 1980s]. There were more problems [with travelling] earlier: we had to sit and wait for a plane for weeks. And now you can travel anywhere. But, of course, in the winter a lot of people come. There are lots of cars: youth from Yuktali drive here day and night. (Interview: GAA, Ust’‐Nyukzha, 2017)

Remarkably, not one of the interviewed residents referring to the story of the missing bridge personally participated in that legendary meeting in the 1980s where the decision against the bridge was supposedly made. Yet, no one considers that decision ‘wrong’, as it is still bolstered with arguments against a year‐round invasion of cars, alcohol, drugs and crime from Yuktali, which takes place when the river is frozen.

We didn't want the bridge ourselves. Everyone was against it because we didn't want too many outsiders, too many people. We thought it would create a mess in the village…. And now they organised a public boat: 40 rubles per trip, from 6 am to 8 or 9 pm. No problem to get to Yuktali. (Interview: GAA, Ust’‐Nyukzha, 2017)

Remoteness as a symbolic and social category tends to be seen as positive and as a kind of protection of indigenous Evenki culture against influences from the *bamovtsy*. In Tynda, we met an indigenous leader, activist and native language teacher. She was born and spent most of her life in Ust’‐Nyukzha and is among the few interlocutors who recall details about the protests against the bridge. In her interpretation, the decision against the bridge was made because the older generation of Evenki residents were equally concerned about both the destruction of reindeer pastures and cultural assimilation. In her mind, the missing bridge might have ensured the retention of Evenki traditional activities and culture in Ust’‐Nyukzha:

In Pervomayskoe that was close [to the BAM], we observe that they immediately lost reindeer herding and fur farming because of the impacts of the nearby BAM construction site. And we [Ust’‐Nyukzha] have more or less preserved ourselves because we were remote from the construction. The railway station is on the one side and we are – on the other. (Interview: KSA, Tynda, 2016)

Evenki cultural centres and indigenous culture organisations at local and regional levels play active roles in the construction and representation of ‘authentic’ indigenous culture in cultural events and development projects with a focus on ethno‐tourism. The largest Evenki festival ‘Bakaldyn’ was hosted by Ust’‐Nyukzha in 2012 and included a number of competitions, ethnic sports, workshops in handicrafts and storytelling, an art exhibition, as well as a conference on indigenous issues (Ermakov 2012). Recently, the festival has grown from an interregional event into an international project that initiated cross‐border cultural exchanges between Evenki communities in the Russian Far East and in China.

Remoteness and indigeneity in Ust’‐Nyukzha are also co‐constructed in reports, development programmes and tourism investment projects in Tyndinskiy District. Such documents are often illustrated with photos of ‘unspoiled’ nature and of Evenki people in national costumes, emphasising the otherness of Evenki culture. Northern regions have a long history of being represented as wilderness and ignoring their built environments (Schweitzer *et al*. 2017).

Finally, mass media outlets also are using the bridge story in Ust’‐Nyukzha to connect remoteness and indigeneity. Not surprisingly, the community has been featured by regional and national media for its successes in promoting indigenous education, culture and traditional industries (e.g. Ermakov 2012). Journalistic reports have repeatedly emphasised the spatial disconnection and isolation of Ust’‐Nyukzha because of the missing bridge (Ermakov 2016), thereby constructing remoteness as exotic otherness of the local community (cf. Mankova 2018). Similar to Luo, Oakes and Schein (this issue), we observe that certain notions of remoteness can be produced and reproduced, as they are seen as a resource that is promising gain. In this process, media organisations, agencies responsible for cultural revitalisation and tourism programmes, as well as indigenous leaders play key roles.

## Discussion and concluding remarks

The ideological shift from a modernist vision of remoteness as a defect to one where ‘hard to access’ and ‘cultural distinction’ are seen as positive is not uniform or unidirectional. The diversity of visions of remoteness found among different groups of interlocutors in Ust’‐Nyukzha challenges simplistic dichotomies such as emic/etic, insider/outsider, Evenki/BAM builders, despite a tangible dividing line between indigenous and non‐indigenous groups. Evenki residents in Ust’‐Nyukzha and elsewhere are not mere victims of state modernisation but stakeholders in regional and global development processes. The non‐existent bridge is a strong symbol for everyone involved: it is as much a potential project and a promise of connectivity that should bridge the gap between two cultural environments as it is a sign of the latent but enduring resistance to sweeping top‐down assimilation. Finally, the missing bridge more recently has become a symbol of state withdrawal resulting in a kind of return of remoteness. No matter what actually led to not building the bridge in the 1980s, everyone agrees that no one would finance it today.

The story told here cannot be generalised for all of Siberia or other ‘remote’ regions. Ust’‐Nyukzha is a special case in many respects, including the fact that its ‘insistence on remoteness’ reaches back into Soviet times, when speaking up against modernisation plans was less common and riskier than in post‐Soviet times. The beginning of the story of the missing bridge also brings us back to the rise of the indigenous rights movement in Russia. Indigenous rights entered local discourses during the 1990s, when global movements entered the previously closed space of the Soviet Union and its successor states. While indigenous leaders, herders and village residents can no longer voice their opinions as openly today, ‘culture’ remains an area where a certain amount of autonomy seems possible. At the same time, remoteness is seen by some as being conducive towards the conservation and development of the indigenous culture. As we have seen, some indigenous leaders connect the infrastructural isolation of Ust’‐Nyukzha with the distinctiveness of Evenki culture. Thus, the ‘moral right to remoteness’ is an implicit one, based on cultural rights which ought to guarantee the preservation of ‘traditional’ ways of life and cultural distinctiveness.

Deliberations about remoteness as *a resource* also emerge in discussions about the prospects for cultural and ecological tourism, the commercialisation of traditional activities and other alternative development projects in local administrations. One could say that the commodification of culture that Comaroff and Comaroff (2009) talk about might be the intended or unintended end goal of equating remoteness and cultural vitality, although current levels of tourism development are still negligible. The example of Ust’‐Nyukzha also reminds us that opposing a bridge is not necessarily a statement against development and connectivity. Here, notions of socio‐spatial remoteness as being something positive coexist with visions about more contacts with other Evenki groups and increased tourist traffic. Despite the fact that Ust’‐Nyukzha's initial statement against infrastructural connectivity was made more than 30 years ago, it is possible to detect an increase in positive attitudes toward spatial remoteness and disconnection there and in other communities of the BAM region. At the same time, the uneven infrastructural and socio‐economic development of the region under post‐Soviet conditions puts a question mark behind future development plans, whether locally desirable or not. Thus, the future might hold an involuntary ‘return to remoteness’ that is separate from a ‘right to remoteness’.

## Notes

1 BAM is the acronym for ‘Baikal–Amur Mainline’; the history and specifics of this railway line will be discussed below.

2 This simplifying description and rushed overview do not give sufficient attention to important contributions of the 1990s, such as Tsing (1993) and Piot (1999). Both grapple with ‘marginality in an out‐of‐the‐way place’, that is with an anthropology of remoteness within a discipline confronting postmodernism, globalisation and transnationalism.

3 This paper is based on fieldwork conducted in the central BAM Region, including the cities of Tynda, Novaya Chara and Severobaykal'sk and the villages of Pervomayskoe, Ust’‐Nyukzha, Ust’‐Urkima, Chapo‐Ologo, Kyust’‐Kemda and Kholodnoe in Amurskaya Province, Zabaykal'skiy Region and the Republic of Buryatiya in 2013, 2016 and 2017.

4 The term was officially introduced during the Soviet period to refer to settlements with a predominant or significant indigenous population.

5 Early Soviet modernisation policies towards indigenous peoples of the North were aimed at bringing them ‘to the next stage of development’ and included a series of campaigns, from collectivisation and agricultural reforms to ‘enlightenment’ and ‘cultural construction’ in the 1930–1950s. The early Soviet policies irreversibly changed traditional lifestyle and culture of the local Evenki: nomadic reindeer herders and hunters, especially women, were pushed out of the taiga and sedentarised into villages with a predominant indigenous population.

6 Our fieldwork in Ust’‐Nyukzha was preceded by long‐term anthropological study among indigenous communities in the northern Zabaykal'skiy Region and Amurskaya Province. One of the authors first learned about ‘the special case of Ust’‐Nyukzha’ in 2013 in Tynda and later in the indigenous villages Pervomayskoe and Ust’‐Urkima, where the story about Ust’‐Nyukzha has been circulating. During a stay in the village in July 2017, a series of community meetings and in‐depth interviews with indigenous people (reindeer herders, education and cultural specialists, activists) and Soviet‐time migrants to the village were conducted. In the course of interviews, special attention was paid to local concepts of remoteness (in relation to connectivity, mobility and indigenous culture) and attitudes to the story of the missing bridge.

7 The case of Ust’‐Nyukzha brings to mind anthropology's most famous ‘bridge study’, namely Gluckman's ‘analysis of a social situation’ (Gluckman 1940). For us, the events surrounding the non‐event of not building a bridge are out of temporal reach; instead our interests focus entirely on how this form of disconnection is interpreted today.

8 The mass outmigration from the BAM region is a post‐Soviet trend that has been visible in most of the settlements located along the railroad with predominantly *bamovtsy* residents.

9 The term ‘Big Land’ (Russ. *bol'shaya zemlya*) is a widely used expression drawing on a popularly imagined juxtaposition between centrally located places and isolated, hard‐to‐access communities (especially in northern Russia).

## References

[soca12648-bib-0001] Arce A. and Long N. (eds.) 2000 Anthropology, development and modernities: exploring discourses, counter‐tendencies and violence. London: Routledge.

[soca12648-bib-0002] Ardener, E. 1987 ‘Remote areas’: some theoretical considerations, in Jackson A. (ed.), Anthropology at home, 38–54. London: Tavistock.

[soca12648-bib-0003] Ardener, E. 2012 ‘Remote areas: some theoretical considerations’, HAU: Journal of Ethnographic Theory 2: 519–33.

[soca12648-bib-0004] Attoh, K. A. 2011 ‘What *kind* of right is the right to the city?’, Progress in Human Geography 35: 669–85.

[soca12648-bib-0005] Campbell, C. 2003 ‘Contrails of globalization and the view from the ground: an essay on isolation in east‐central Siberia’, Polar Geography 27: 97–120.

[soca12648-bib-0006] Chu, Y. Y. 2014 ‘When infrastructures attack: the workings of disrepair in China’, American Ethnologist 41: 351–67.

[soca12648-bib-0007] Comaroff, J. L. and J. Comaroff 2009 Ethnicity, Inc. Chicago, IL: The University of Chicago Press.

[soca12648-bib-0008] Ermakov, M. 2012 V Tyndinskom rayone otgremel fol'klorno‐etnograficheskiy forum ‘Bakaldyn‐2012’. Dal'nevostochnoe Informatsionnoe Agentstvo ‘Port Amur’ (https​://porta​mur.ru/news/detai​l/v-tyind​inskom-rayone-otgre​mel-folkl​orno-etnog​rafic​heskiy-forum-bakal​dyin-2012/?print​=Y) Accessed 26 November 2017.

[soca12648-bib-0009] Ermakov, M. 2016 Osen'yu tyndinskuyu Ust’‐Nyukzhu s ‘Bol'shoy Zemley’ svyazhet puma (https​://www.ampra​vda.ru/2016/04/02/65570.html) Accessed 26 November 2017.

[soca12648-bib-0010] Ferguson, J. 1994 The anti‐politics machine: development, depoliticization and bureaucratic power in Lesotho. Minneapolis, MN: University of Minnesota Press.

[soca12648-bib-0011] Fondahl, G. , O. Lazebnik , G. Poelzer and V. Robbek 2001 ‘Native “land claims”, Russian style’, Canadian Geographer/Geographe Canadien 45: 545–61.

[soca12648-bib-0012] Gellner D. N. (ed.) 2007 Resistance and the state: Nepalese experiences. New York: Berghahn Books.

[soca12648-bib-0013] Ghosh, K. 2006 ‘Between global flows and local dams: indigenousness, locality, and the transnational sphere in Jharkhand, India’, Cultural Anthropology 21: 501–34.

[soca12648-bib-0014] Gluckman, M. 1940 ‘Analysis of a social situation in modern Zululand’, Bantu Studies 14: 1–30.

[soca12648-bib-0015] Grant, B. 1995 In the Soviet house of culture: a century of perestroikas. Princeton, NJ: Princeton University Press.

[soca12648-bib-0016] Hall, S. 1996 Introduction: Who needs identity, in Hall S. and du Gay P. (eds.), Questions of cultural identity, 1–17. Los Angeles, CA: SAGE.

[soca12648-bib-0017] Harms, E. , S. Hussain and S. Shneiderman 2014 ‘Remote and edgy: new takes on old anthropological themes’, HAU: Journal of Ethnographic Theory 4: 361–81.

[soca12648-bib-0018] Harvey, D. 2008 ‘The right to the city’, New Left Review 53: 23–40.

[soca12648-bib-0019] Harvey, P. and H. Knox 2012 ‘The enchantments of infrastructure’, Mobilities 7: 521–36.

[soca12648-bib-0020] Humphrey, C. 1983 Karl Marx collective: economy, society and religion in a Siberian collective farm. Cambridge: Cambridge University Press.

[soca12648-bib-0021] Humphrey, C. 2003 Rethinking infrastructure: Siberian cities and the great freeze of January 2001, in Schneider J. and Susser I. (eds.), Wounded cities, 91–107. Oxford: Berg.

[soca12648-bib-0022] Hussain, S. 2015 Remoteness and modernity: transformation and continuity in northern Pakistan. New Haven, CT: Yale University Press.

[soca12648-bib-0023] Kaschuba, W. 2004 Die Überwindung der Distanz. Zeit und Raum in der europäischen Moderne. Frankfurt/Main: Fischer.

[soca12648-bib-0024] Kuklina, V. and E. C. Holland 2018 ‘The roads of the Sayan Mountains: theorizing remoteness in eastern Siberia’, Geoforum 88: 36–44.

[soca12648-bib-0025] Lavrillier, A. 2013 ‘Anthropology and applied anthropology in Siberia: questions and solutions concerning a nomadic school among Evenk reindeer herders, in Kasten E. and de Graaf T. (eds.), Cultural heritage, 105–27. Fürstenberg‐Havel: Kulturstiftung Sibirien.

[soca12648-bib-0026] Lefebvre, H. 1996 [1968]. The right to the city, in Kofman E. and Lebas E. (eds.), Writings on cities, 146–59. Cambridge, MA: Wiley‐Blackwell.

[soca12648-bib-0027] Li, T. M. 2000 ‘Articulating indigenous identity in Indonesia: resource politics and the tribal slot’, Comparative Studies in Society and History 42: 149–79.

[soca12648-bib-0028] Li, T. M. 2007 The will to improve: governmentality, development, and the practice of politics. Durham, NC: Duke University Press.

[soca12648-bib-0029] Li, T. M. 2017 ‘After development: surplus population and the politics of entitlement’, Development and Change 48: 1247–61.

[soca12648-bib-0030] Mankova, P. 2018 ‘Making sense of the remote areas: films and stories from a tundra village’, Sibirica 17: 60–84.

[soca12648-bib-0031] Pasport 1990 Pasport Tyndinskogo rayona Amurskoy oblasti, 1985–1990. Tynda: Arkhiv administratsii Tyndinskogo rayona.

[soca12648-bib-0032] Pasport 2012 Pasport munitsipal'nogo obrazovaniya ‘Ust’‐Nyukzhinskiy sel'sovet’. Ust’‐Nyukzha: Arkhiv administratsii Ust’‐Nyukzhi.

[soca12648-bib-0033] Pasport 2017a Pasport munitsipal'nogo obrazovaniya ‘Ust’‐Nyukzhinskiy sel'sovet’. Ust’‐Nyukzha: Arkhiv administratsii Ust’‐Nyukzhi.

[soca12648-bib-0034] Pasport 2017b Pasport munitsipal'nogo obrazovaniya ‘Yuktalinskiy sel'sovet’. Ust’‐Nyukzha: Arkhiv administratsii Ust’‐Nyukzhi.

[soca12648-bib-0035] Pika A. (ed.) 1999 Neotraditionalism in the Russian North: indigenous peoples and the legacy of perestroika. Edmonton: Canadian Circumpolar Institute Press.

[soca12648-bib-0036] Piot, C. 1999 Remotely global: village modernity in West Africa. Chicago, IL: The University of Chicago Press.

[soca12648-bib-0037] Povoroznyuk, O. A. 2011 Zabaykal'skie evenki: sotsial'no‐ekonomicheskie i kul'turnye transformatsii XX–XXI vekakh. Moscow: Institut etnologii i antropologii im. N.N. Miklukho‐Maklaya, Rossiyskaya Akademiya Nauk.

[soca12648-bib-0038] Rainey, T. B. 1991 ‘Siberian writers and the struggle to save Lake Baikal’, Environmental History Review 15: 46–60.

[soca12648-bib-0039] Saxer, M. 2016 ‘Pathways: a concept, field site and methodological approach to study remoteness and connectivity’, HIMALAYA, the Journal of the Association for Nepal and Himalayan Studies 36: 104–19.

[soca12648-bib-0040] Schivelbusch, W. 2000 Geschichte der Eisenbahnreise. Zur Industrialisierung von Raum und Zeit im 19. Jahrhundert. Frankfurt/Main: Fischer.

[soca12648-bib-0041] Schüler, S. 2017 ‘The zone à defendre of Notre‐Dame‐des‐Landes in France: an ambivalent space for social critique’, Urbanities 7: 45–62.

[soca12648-bib-0042] Schweitzer, P. , O. Povoroznyuk and S. Schiesser 2017 ‘Beyond wilderness: towards an anthropology of infrastructure and the built environment in the Russian North’, The Polar Journal 7: 58–85. 29098112 10.1080/2154896X.2017.1334427 PMC5632953

[soca12648-bib-0043] Scott, J. C. 1998 Seeing like a state: how certain schemes to improve the human condition have failed. New Haven, CT: Yale University Press.

[soca12648-bib-0044] Scott, J. C. 2009 The art of not being governed: an anarchist history of upland southeast Asia. New Haven, CT: Yale University Press.

[soca12648-bib-0045] Simpson, A. 2014 Mohawk interruptus: political life across the borders of settler states. Durham, NC: Duke University Press.

[soca12648-bib-0046] Simpson, A. 2017 ‘The ruse of consent and the anatomy of “refusal”: cases from indigenous North America and Australia’, Postcolonial Studies 20: 18–33.

[soca12648-bib-0047] Ssorin‐Chaikov, N. 2016 ‘Soviet debris: failure and the poetics of unfinished construction in northern Siberia’, Social Research 83: 689–721.

[soca12648-bib-0048] Tsing, A. L. 1993 In the realm of the diamond queen: marginality in an out‐of‐the‐way place. Princeton, NJ: Princeton University Press.

[soca12648-bib-0049] Tugolukov, V. A. 2005 O rezul'tatakh obsledovaniya evenkiyskikh kolkhozov Amurskoy oblasti, in Sokolova Z. P. and Pivneva E. A. (eds.), Etnologicheskaya ekspertiza. Narody Severa Rossii 1959–1962 gody, 228–36. Moscow: IEA RAS.

[soca12648-bib-0050] Turaev, V. A. 2004 ‘Evenki Amurskoy oblasti: sotsial'no‐demograficheskie i etnokul'turnye protsessy (po rezul'tatam polevykh issledovanii 2003 g.)’, Vestnik DVO RAN 2.

[soca12648-bib-0051] von Schnitzler, A. 2016 Democracy's infrastructure: techno‐politics and protest after apartheid. Princeton, NJ: Princeton University Press.

[soca12648-bib-0052] Ward, C. J. 2009 Brezhnev's folly: the building of BAM and late Soviet socialism. Pittsburgh, PA: University of Pittsburgh Press.

[soca12648-bib-0053] Zaharchenko, T. 1990 ‘The environmental movement and ecological law in the Soviet Union: the process of transformation’, Ecology Law Quarterly 17: 454–75.

[soca12648-bib-0054] Ziegler, C. E. 1987 Environmental policy in the USSR. Amherst, MA: The University of Massachusetts Press.

